# Cell Type-Specific Transcriptome Profiling Reveals a Role for Thioredoxin During Tumor Initiation

**DOI:** 10.3389/fimmu.2022.818893

**Published:** 2022-02-17

**Authors:** Benjamin G. Korte, Morgan A. Giese, Gayathri Ramakrishnan, Stella Ma, David Bennin, Julie Rindy, Colin N. Dewey, Anna Huttenlocher

**Affiliations:** ^1^ Department of Pediatrics and Medical Microbiology and Immunology, University of Wisconsin – Madison, Madison, WI, United States; ^2^ Cancer Biology Graduate Program, University of Wisconsin – Madison, Madison, WI, United States; ^3^ Cellular and Molecular Biology Graduate Program, University of Wisconsin – Madison, Madison, WI, United States; ^4^ Department of Biostatistics and Medical Informatics, University of Wisconsin – Madison, Madison, WI, United States; ^5^ Department of Pediatrics, University of Wisconsin – Madison, Madison, WI, United States

**Keywords:** neutrophil, migration, tumor initiation, thioredoxin (txn), gene expression, keratinocyte

## Abstract

Neutrophils in the tumor microenvironment exhibit altered functions. However, the changes in neutrophil behavior during tumor initiation remain unclear. Here we used Translating Ribosomal Affinity Purification (TRAP) and RNA sequencing to identify neutrophil, macrophage and transformed epithelial cell transcriptional changes induced by oncogenic Ras^G12V^ in larval zebrafish. We found that transformed epithelial cells and neutrophils, but not macrophages, had significant changes in gene expression in larval zebrafish. Interestingly, neutrophils had more significantly down-regulated genes, whereas gene expression was primarily upregulated in transformed epithelial cells. The antioxidant, thioredoxin (*txn*), a small thiol that regulates reduction-oxidation (redox) balance, was upregulated in transformed keratinocytes and neutrophils in response to oncogenic Ras. To determine the role of thioredoxin during tumor initiation, we generated a zebrafish thioredoxin mutant. We observed an increase in wound-induced reactive oxygen species signaling and neutrophil recruitment in thioredoxin-deficient zebrafish. Transformed keratinocytes also showed increased proliferation and reduced apoptosis in thioredoxin-deficient larvae. Using live imaging, we visualized neutrophil behavior near transformed cells and found increased neutrophil recruitment and altered motility dynamics. Finally, in the absence of neutrophils, transformed keratinocytes no longer exhibited increased proliferation in thioredoxin mutants. Taken together, our findings demonstrate that tumor initiation induces changes in neutrophil gene expression and behavior that can impact proliferation of transformed cells in the early tumor microenvironment.

## Introduction

Neutrophils respond to tissue damage cues and are actively recruited to the tumor microenvironment (TME) (1, 2). Although neutrophils can have either pro- or anti-tumor effects (3), the presence of neutrophils in tumors often correlate with poor prognosis (4, 5). Therefore, there is increasing interest in understanding how neutrophils are recruited to and interact with transformed cells in the developing tumor microenvironment.

The zebrafish larval model allows for unparalleled non-invasive imaging of the early stages of tumor initiation and interactions with innate immune cells. Zebrafish larvae have a functional innate immune system comprised of macrophages and neutrophils by 2 days post-fertilization (dpf). This early developmental window allows the investigation of specific interactions between innate immune cells and the tumor-initiating niche. Neutrophils are highly migratory cells that move within interstitial tissues near transformed cells. *In vivo* studies in zebrafish have shown that neutrophils are recruited shortly after oncogene-induced transformation (6, 7). Furthermore, recruited neutrophils make frequent, direct cell-cell contact with HRas^G12V^-transformed cells, occasionally forming long membrane tethers between cells (6). At this early stage in tumorigenesis, neutrophils support proliferation and epithelial to mesenchymal transition of transformed epithelial and glial cells (8, 9). Additionally, recent evidence suggests that neutrophil migratory behavior changes during the course of tumor development in mouse models, with early stage tumors inducing migration of bone marrow-derived neutrophils and late stage tumors inducing a slower, immunosuppressive neutrophil migration (10). Therefore, the crosstalk between neutrophils and tumor cells is dynamic during tumor progression.

Several signaling pathways are involved in neutrophil chemotaxis and recruitment, many of which are also upregulated in the TME. One of the primary chemotactic pathways that regulates neutrophil migration is the CXCL8 (IL8)-CXCR1/2 pathway. CXCL8 is upregulated in many cancers including solid tumors (brain, breast, colon, gastric, lung, and others) and blood cancers (AML, CLL, Hodgkin’s lymphoma) and high levels of CXCL8 expression are often linked with disease progression (11). Reactive oxygen species (ROS) are another potent neutrophil chemoattractant produced in transformed cells (6) and tumors (12). Studying neutrophil migratory behavior early in tumorigenesis may provide insight into how these cells elict their tumor-supporting effects.

To identify candidate genes that regulate neutrophil behavior in the TME, we conducted tissue-specific RNA-sequencing of HRas^G12V^-transformed keratinocytes, neutrophils, and macrophages in zebrafish larvae using translating ribosome affinity purification (TRAP)-seq (13, 14). We found that the anti-oxidant thioredoxin was increased approximately five-fold in HRas^G12V^-transformed keratinocytes and three-fold in neutrophils, over wildtype HRas-expressing zebrafish. We generated thioredoxin mutants that showed increased neutrophil recruitment and altered neutrophil motility behavior near transformed cells. These mutants also exhibited enhanced neutrophil-dependent proliferation of transformed cells, suggesting that thioredoxin modulates neutrophil behavior and early tumor progression.

## Results

### Oncogenic Ras^G12V^ Induces Differentially Expressed Genes in Neutrophils and Transformed Cells in Zebrafish Larvae

**Figure 1:**
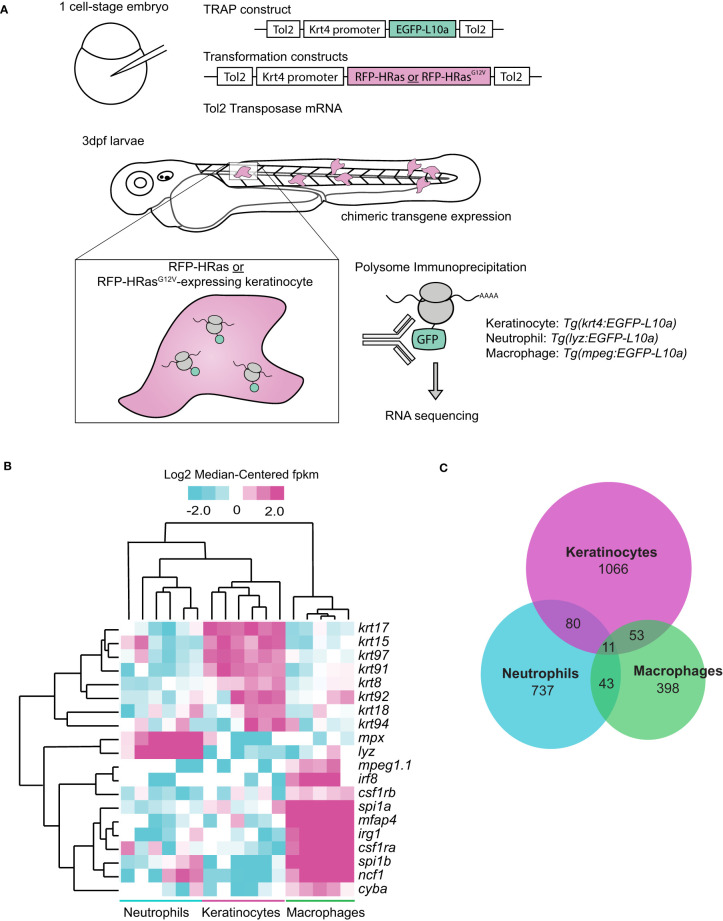
Oncogenic HRas induces differentially expressed genes in neutrophils and transformed cells in zebrafish larvae. **(A)** Schematic of Translating Ribosomal Affinity Purification (TRAP) procedure. Wild-type embryos were injected with the TRAP plasmid (pTol2-Krt4-EGFP-L10a) and either control (pTol2-Krt4-HRas-mcherry) or oncogenic transformation (pTol2-Krt4-HRas^G12V^-mcherry) constructs. Immunoprecipitation and sequencing were conducted 3dpf on batches of ~50 larvae collected on three separate days (n=3). *Tg(LyzC : EGFP-L10a)* or *Tg(mpeg:EGFP-L10a)* larvae were injected with either control (pTol2-Krt4-HRas-mcherry) or oncogenic Ras (pTol2-Krt4-HRas^G12V^-mcherry) constructs for neutrophil- or macrophage-targeted L10a expression, respectively. **(B)** Cell type specific genes expressed in keratinocytes (*krt17, krt15, krt97, krt91, krt8, krt92, krt18*, krt94), neutrophils (*mpx,lyz*), or macrophages (*mpeg1.1, irf8, csf1rb, spi1a, mfap4, irg1, csf1ra, spi1b, ncf1, cyba*) and their expression profiles in TRAP samples from keratinocytes, neutrophils, and macrophages. **(C)** Venn diagram showing distribution of genes altered >2fold by TRAP-seq in response to HRas^G12V^ expressing keratinocytes (n=3).

We sought to identify changes in gene expression that regulate leukocyte behavior in zebrafish larvae in response to expression of oncogenic HRas^G12V^ in keratinocytes. We performed translating ribosome affinity purification (TRAP) (13, 15) and subsequent RNA sequencing of three cell-types (Ras-expressing keratinocytes, neutrophils, and macrophages) in 3 days post-fertilization (dpf) larvae in response to wild type (WT) or oncogenic HRas^G12V^ (16). Briefly, keratinocytes co-expressed either control HRas (pTol2-Krt4-RFP-HRas) or constitutively-active HRas^G12V^ (pTol2-Krt4-RFP-HRas^G12V^) and EGFP-tagged ribosomal subunit L10a using the Krt4 promoter (**Figure 1A**). To identify gene expression changes within transformed cells, GFP-tagged ribosomes were immunoprecipitated and their associated transcripts were sequenced (**Figure 1A**). Neutrophil or macrophage-specific ribosomes were also isolated from whole larvae using transgenic lines *Tg(Lyzc : EGFP-L10a)* or *Tg(mpeg:EGFP-L10a)* to identify differentially expressed genes in neutrophils and macrophages, respectively (**Figure 1A**). Sequencing confirmed expression of known cell-type-specific genes for neutrophils, macrophages, and keratinocytes in the analyzed samples, providing validation for the method (**Figure 1B**). We identified genes altered >2fold in response to HRas^G12V^ expressing keratinocytes for all three cell types (**Figure 1C**). A small number of transcripts overlapped between cell types, indicative of a cell-type specific response to HRas-transformed keratinocytes.

**Figure 2:**
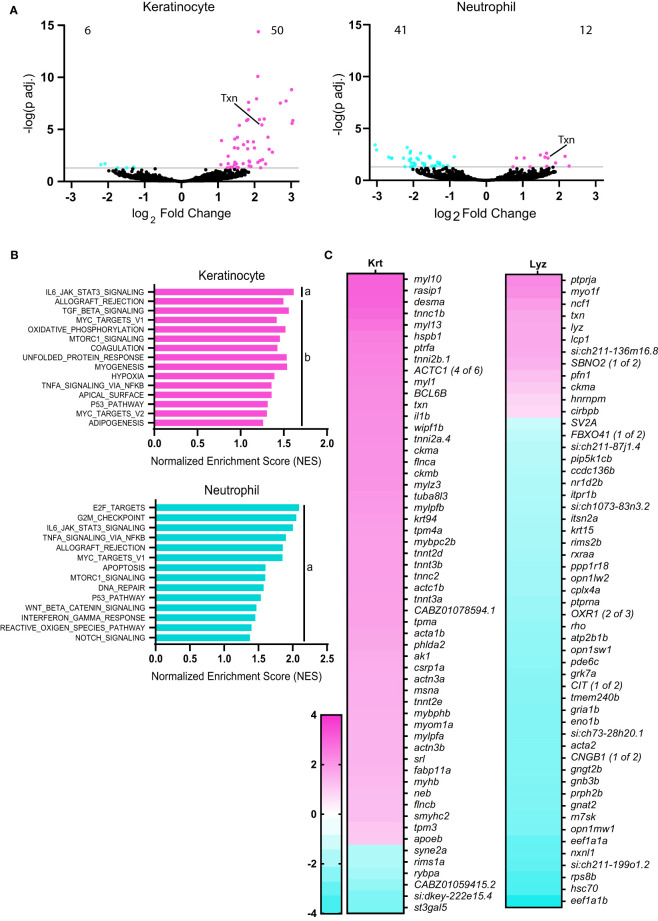
Thioredoxin expression is induced in neutrophils and keratinocytes with HRas^G12V^ transformation. **(A)** Volcano plots displaying number of significantly (adjusted p<0.05) downregulated and upregulated genes in keratinocytes and neutrophils in HRas^G12V^-keratinocyte expressing zebrafish larvae, compared to wild-type HRas controls. **(B)** Gene Set Enrichment Analysis (GSEA) of significantly enriched Hallmark pathways in neutrophils and keratinocytes for HRas^G12V^-keratinocyte expressing zebrafish. FDR q-value is (a) below 0.10 or (b) 0.10-0.25. **(C)** Heat maps displaying significantly differentially expressed genes (adjusted p<0.05) in keratinocytes (56 genes) and neutrophils (53 genes).

We identified significantly deferentially expressed genes in transformed keratinocytes (56 genes) and neutrophils (53 genes), but not in macrophages (**Figure 2A**). As expected, gene expression was upregulated in transformed keratinocytes, with the majority of transcripts showing increased expression. In contrast, the majority of the differentially expressed genes in neutrophils showed reduced expression. While neutrophils collected from HRas-transformed keratinocyte larvae showed differential gene expression, macrophages did not. The significant changes in gene expression observed in neutrophils, may be due to neutrophil trafficking around transformed keratinocytes (7).

### Thioredoxin Expression Is Induced in Neutrophils and Keratinocytes With HRas^G12V^ Transformation

Gene set enrichment analysis (GSEA) revealed significant enrichment of the IL6-Jak-Stat3-signaling and Tnfα-signaling-via-NF-κB in transformed cells and neutrophils. There was also enrichment in reactive-oxygen-species signaling pathways in neutrophils in response to oncogenic Ras (**Figure 2B**). Hierarchical clustering indicates overlapping enrichment of many Hallmark pathways between neutrophils and keratinocytes (**Figure S1A**).To further characterize the role of ROS signaling on leukocyte behavior near transformed cells, we focused on the role of the ROS regulator and antioxidant thioredoxin. Thioredoxin (*txn*) was among the most highly upregulated genes in keratinocytes in response to oncogenic Ras (**Figure 2C**) and was also found to be significantly upregulated in neutrophils. This finding is consistent with human *in vitro* studies showing that oncogenic Ras-transformation induces several antioxidant genes (17). In addition, thioredoxin expression was upregulated in ten of thirteen human carcinoma samples based on bulk sequencing available through The Cancer Genome Atlas (TCGA), as compared to healthy tissue (**Figure S1B**) (51). Furthermore, human tumors are known to produce ROS above normal tissue levels (12), and thus thioredoxin may play an important role in cellular redox balance during tumorigenesis to reduce ROS levels.

### Thioredoxin Regulates Neutrophil Recruitment and Redox Balance in Damaged Tissues

**Figure 3:**
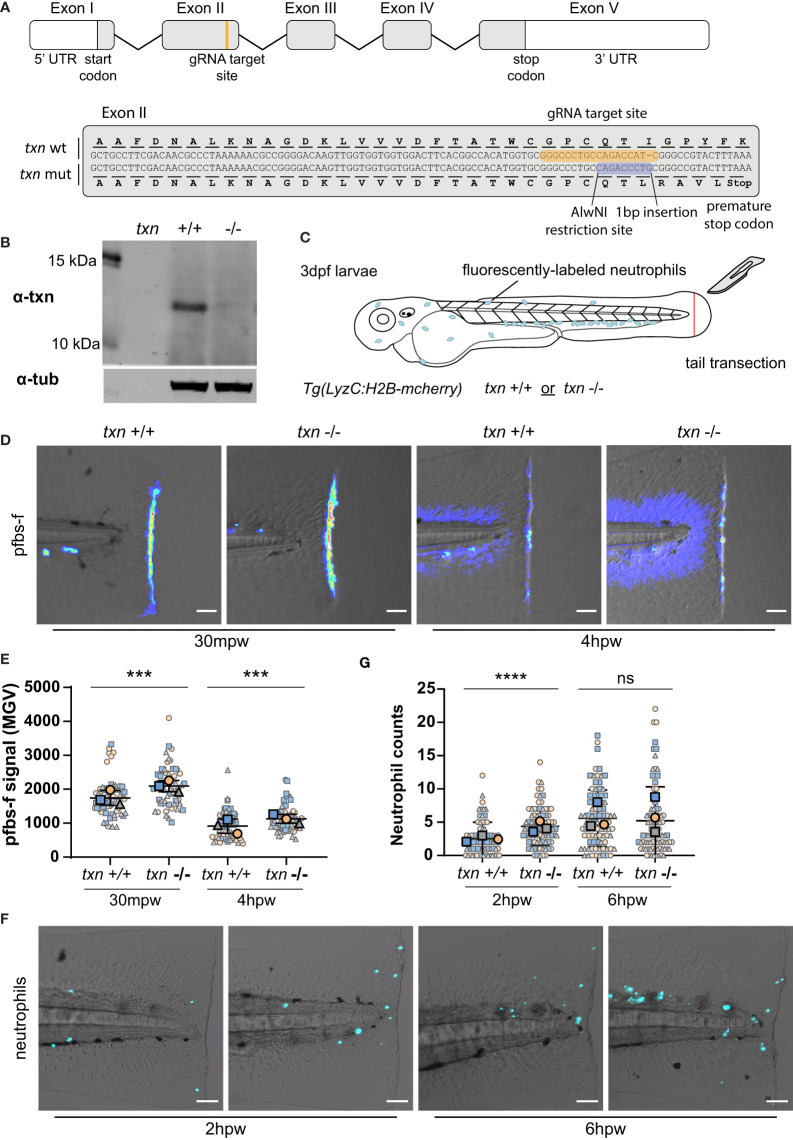
Thioredoxin regulates neutrophil recruitment and redox balance in damaged tissues. **(A)** Schematic of *txn* gene with annotations of gRNA target site and 1bp insertion identified in txn mutant larvae. **(B)** Western blot of Txn and αTubulin on whole lysates from *txn* wildtype and mutant larvae. **(C)** Schematic of tail transection assay to measure neutrophil recruitment and H_2_O_2_ abundance conducted on *txn* ^+/+^ Tg(*LyzC*:*H2B-mcherry*) or *txn* ^-/-^Tg(*LyzC*:*H2B-mcherry*) larvae 3dpf. **(D)** pfbs-f probe for H_2_O_2_ (1uM) in response to tail transection 30mpw (wt n=56, mut n=54) and 4hpw (wt n=51, mut n=51) with **(E)** quantification of signal density in region posterior to notochord. **(F)** Neutrophil recruitment in response to tail transection 2hpw (wt n=79, mut n=85) and 6hpw (wt n=87, mut n=90) with **(G)** quantification of neutrophil abundance in region posterior to notochord. Scale bar, 50um. Large, bolded shapes indicate average value per replicate (n), with small shapes representing data points from independent larvae. Samples were analyzed for statistical significance by Mann-Whitney U test (p < 0.001***, p < 0.0001****). ns, not significant.

To characterize the role of thioredoxin during tumor initiation, we generated a *thioredoxin (txn)* mutant using CRISPR/Cas9 gene editing. Despite its known role as an antioxidant, the role of thioredoxin during inflammation is unclear with both pro- and anti-inflammatory functions reported (18–21). We generated a zebrafish *txn* mutant harboring a 1bp insertion leading to a premature stop codon (**Figure 3A**). Western blotting of 3dpf larval lysates showed loss of the full-length protein in *txn* mutants (**Figure 3B**). *txn* ^-/-^ larvae appeared morphologically normal and developed similar neutrophil numbers compared to wildtype larvae (**Figures S2A, B**), suggesting that thioredoxin is not required for normal development.

Previous studies have shown that a gradient of H_2_O_2_ is generated early after wounding and regulates neutrophil recruitment (22–24). To determine if thioredoxin alters ROS signaling and neutrophil recruitment after tissue damage, we wounded control and thioredoxin-deficient zebrafish by tail transection at 72 hours post fertilization (hpf) (**Figure 3C**). We assayed H_2_O_2_ abundance using the fluorescent H_2_O_2_ sensor, pentafluorobenzene sulphonyl fluorescein (pfbs-f) and found that *txn* mutant larvae displayed increased H_2_O_2_ accumulation both 30 minutes and 4 hours post-wound (hpw) (**Figures 3D, E**). These findings suggest that thioredoxin regulates the redox balance of damaged tissues and that its deletion induces a more robust gradient of H_2_O_2_ at the wound edge.

To determine if increased wound-associated ROS altered neutrophil recruitment to wounds, we quantified neutrophil numbers at a wound in control and thioredoxin mutants. We observed a small increase in neutrophil infiltration at 2 but not 6hpw (**Figures 3F, G**), consistent with H_2_O_2_ providing an early recruitment cue (24). To determine if other neutrophil recruitment pathways are altered in thioredoxin mutants, we quantified expression of inflammatory cytokines. Loss of thioredoxin did not affect basal gene expression of inflammatory cytokines, including the neutrophil chemoattractants *cxcl8a and cxcl8b.1* (**Figure S2C**). Taken together, our findings suggest that thioredoxin limits neutrophil recruitment to wounds, likely through the modulation of H_2_O_2_ production.

**Figure 4:**
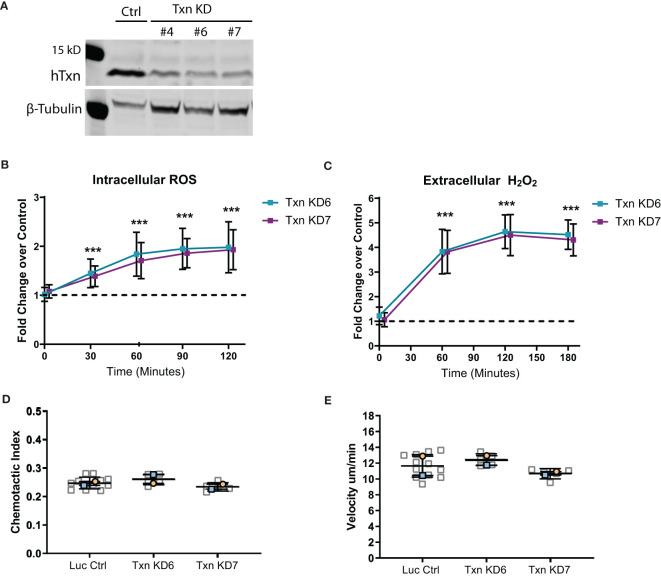
Thioredoxin depletion in a neutrophil-like cell line increases ROS but does not intrinsically affect motility. **(A)** Western blot of thioredoxin confirming CRISPR/Cas9 mediated txn knockdown in three HL60 clonal lines (KD4, KD6 and KD7) compared to luciferase control, with beta-tubulin as a loading control. **(B)** Quantification of intracellular ROS production (Carboxy-H2DCFDA probe) over time in txn knockdown lines compared to control (n=3). **(C)** Quantification of extracellular H_2_O_2_ production (Amplex Red/HRP Probe) over time in txn knockdown lines compared to control (n=3). **(D)** 2D chemotactic index and **(E)** mean track speed for txn knockdown lines compared to control (N = 2 repeats, n = 5-12 devices). Samples were analyzed for statistical significance *via* generalized estimating equation (GEE) fit to the log fold change with confidence intervals set to 95% coverage **(B, C)** or t-test **(D, E)** (p < 0.001***).

Since neutrophils also show an increase in thioredoxin expression in response to transformed cell cues, we next wanted to determine if there were neutrophil-intrinsic changes that may contribute to increased neutrophil recruitment to wounds. To address this question we knocked down thioredoxin in human neutrophil-like HL60 cells using CRISPR/Cas9 gene editing (**Figure 4A**). We found that decreased thioredoxin expression in neutrophil-like cells generated more ROS in response to PMA stimulation, suggesting that thioredoxin has neutrophil intrinsic effects on ROS signaling (**Figures 4B, C**). However, these cells exhibited normal chemotactic migration including similar velocity to wild type cells (**Figures 4D, E**). In accordance with these findings, we also determined that neutrophil random migration speed in the head region of zebrafish larvae was similar between control and mutant, suggesting that intrinsic neutrophil function was not significantly altered in thioredoxin mutants (**Figures S3A, B**).

### Transformed Keratinocytes Exhibit Increased Proliferation and Reduced Apoptosis During Tumor Initiation in Thioredoxin Mutants

Due to very low survival of zebrafish larvae with HRas^G12V^ driven expression of transformed keratinocytes, we modified our model to express oncogenic KRas^G12V^ under the Krtt1c19e basal keratinocyte promoter (**Figure S4**). Increased expression of thioredoxin within KRas^G12V^ transformed keratinocytes was confirmed (data not shown). All experimental data were collected using this KRas-transformed keratinocyte model.

**Figure 5:**
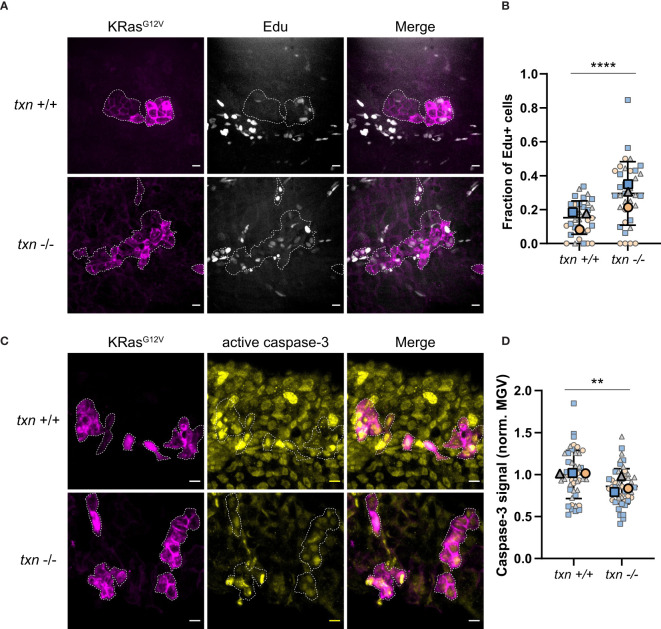
Transformed keratinocytes exhibit increased proliferation and reduced apoptosis during tumor initiation in *thioredoxin* mutants. **(A)** EdU staining (white) of *txn* ^+/+^ or *txn* ^-/-^ larvae with KRas^G12V^-expressing basal keratinocytes (magenta) at 3dpf. **(B)** Quantification of the frequency of EdU-positive KRas^G12V^-expressing basal cells 3dpf in *txn* wt (n=35) and mutant (n=35) larvae. **(C)** Cleaved caspase-3 immunofluorescence of *txn* ^+/+^ or *txn* ^-/-^ larvae. **(D)** Quantification of relative signal intensity within transformed cells 3dpf in *txn* ^+/+^ (n=45) and *txn* ^-/-^ (n=51) larvae. Scale bar, 10um. Large, bolded shapes indicate average value per replicate (n), with small shapes representing data points from independent larvae. Samples were analyzed for statistical significance *via* t-test (p < 0.001***, p < 0.0001****).

Previous studies have demonstrated that neutrophils regulate the proliferation of transformed cells in zebrafish larvae (7, 9). To determine the effect of *txn* on transformed cell fate, proliferation was assessed using EdU incorporation during S-phase. Constitutively-active KRas^G12V^ or WT KRas were expressed in basal keratinocytes in either *txn* ^+/+^ or *txn* ^-/-^ larvae (**Figure S4**). Quantification of EdU incorporation showed increased proliferation of transformed cells in the *txn* mutants, suggesting that thioredoxin inhibits early KRas^G12V^-driven proliferation *in vivo* (**Figures 5A, B**). We also found reduced caspase-3 staining compared to wildtype controls (**Figures 5C, D**) suggesting *txn* promotes transformed cell apoptosis. As loss of thioredoxin affects redox balance in the wounded fin (**Figures 3D, E**), we tested whether changes in redox signaling could be responsible for the proliferative phenotype. Using metabolic imaging, we quantified differences in redox signaling within and around transformed keratinocytes. We identified a trend towards a more oxidative state in *txn* mutants, but this was not statistically significant (data not shown). Our findings indicate that thioredoxin suppresses the transformed cell growth through multiple mechanisms during tumor initiation in zebrafish larvae.

### Thioredoxin Affects Neutrophil Behavior Around KRas^G12V^-Transformed Keratinocytes

**Figure 6:**
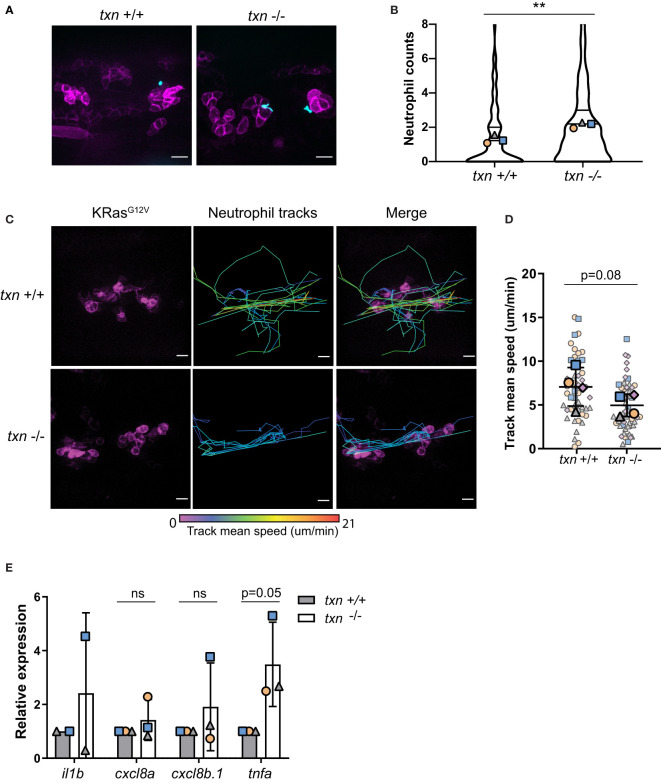
Thioredoxin affects neutrophil motility around KRas^G12V^-transformed keratinocytes. **(A)** Neutrophil (blue) recruitment around KRas^G12V^-expressing basal keratinocytes (magenta) in *txn* ^+/+^ Tg(*LyzC*:*H2B-mcherry*) or *txn* ^-/-^ Tg(*LyzC*:*H2B-mcherry*) larvae 3dpf. **(B)** Quantification of neutrophil abundance in *txn* wt (n=74) and mutant (n=116) larvae. **(C)** Stills from live imaging of neutrophil behavior around KRas^G12V^-expressing cells in *txn* wt and mutant larvae. Neutrophils were tracked across frames (1.5min/frame) and track color delineates instantaneous neutrophil velocity. **(D)** Quantification of average neutrophil speed in *txn* wt (n=4) and mutant (n=4) larvae. **(E)** TRAP-qPCR of KRas^G12V^-expressing genes assessing inflammatory cytokines *il1b*, *cxcl8a*, *cxcl8b.1*, *tnfa* (pool, 30 larvae/replicate, wt n=3, mut n=3). Scale bar, 10uM. Bolded shapes indicate average values per larvae, and small shapes represent instantaneous velocities of individual neutrophils. Samples were analyzed for statistical significance *via* Mann-Whitney U test **(B)** and t-test **(D, E)** (p < 0.01**). ns, not significant.

Cancer has been described as a wound that “does not heal”. Similar to a wound, early innate immune inflammation around transformed cells is mediated by redox signaling (6). In accordance with the wound response (**Figures 3F, G**), there was a small increase in neutrophil abundance around KRas^G12V^-expressing cells in the *txn* mutant larvae as compared to control larvae (**Figures 6A, B**). Additionally, live imaging of neutrophil behavior revealed neutrophils were slower on average around KRas^G12V^-transformed cells in *txn* null larvae (**Figures 6C, D**). To determine if this change in neutrophil behavior in the tumor microenvironment correlated with changes in inflammatory cytokines, we used TRAP and qPCR to analyze cytokine expression in KRas^G12V^-transformed keratinocytes. We found that *tnfα* expression was increased roughly two-fold within transformed cells in *txn* mutant larvae (**Figure 6E**). Basal TNFα expression was unaffected in *txn* mutants compared to WT larvae (**Figure S2C**). Therefore, thioredoxin impairs induction of TNFα inflammatory cytokine production during tumor initiation (**Figure 6E**). No change in keratinocyte gene expression was identified for neutrophil chemoattractants *cxcl8a and cxcl8b.1* in the thioredoxin mutants (**Figure 6E**). Taken together, our findings suggest that thioredoxin inhibits neutrophil recruitment and retention around transformed cells *in vivo*, possibly by regulating the redox status and cytokine production in the tumor microenvironment.

### Transformed Keratinocyte Proliferation Is Not Altered in Thioredoxin Mutants in the Absence of Neutrophils

Since we observed altered neutrophil behavior in thioredoxin-deficient larvae, we then tested the requirement of neutrophils for the increased proliferation of transformed keratinocytes. Neutrophils can produce proliferative factors such as IL-8 to directly promote tumor growth (11). It has been postulated that they can also release growth factors such as EGF, HGF, and PDGF (25).

**Figure 7:**
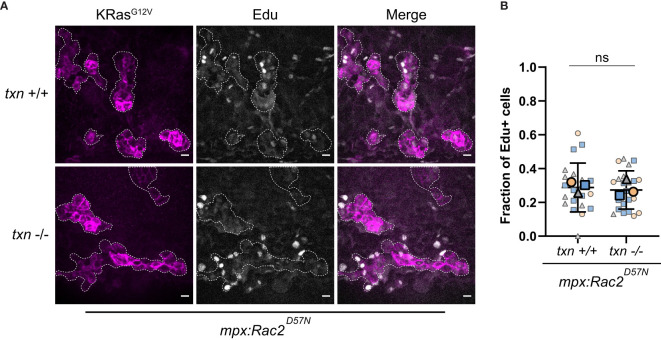
Transformed keratinocyte proliferation is not altered in *thioredoxin* mutants in the absence of neutrophils. **(A)** EdU staining (white) of neutrophil-deficient Tg(*mpx:Rac2^D57N^-mcherry*) *txn* ^+/+^ and Tg(*mpx:Rac2^D57N^-mcherry*) *txn* ^-/-^ larvae with KRas^G12V^-expressing basal keratinocytes (magenta) 3dpf. **(B)** Quantification of frequency of EdU-positive KRas^G12V^-expressing basal keratinocytes 3dpf in *Rac2^D57N^ txn* wt (n=21) and *Rac2^D57N^ txn* mutant (n=22) larvae. Scale bar, 10um. Bolded shapes indicate average value per technical replicate, with shaded values representing data points from independent larvae. Samples were analyzed for statistical significance *via* t-test ns, not significant.

To determine if the presence of neutrophils is necessary for the increase in proliferation in thioredoxin-deficient larvae, we inhibited neutrophil recruitment to transformed cells using an established model with impaired neutrophil function. A transgenic zebrafish line expressing a dominant inhibitory Rac2 mutation (Rac2D57N) in neutrophils renders neutrophils incapable of migrating out of the vasculature (26). In the Rac2D57N background, we found that thioredoxin deficient larvae had no increase in the proliferation of cells expressing KRas^G12V^ compared to WT KRas. Specifically, we found that the frequency of EdU-positive transformed cells between *txn* wildtype and mutant larvae were similar (**Figures 7A, B**). This is in contrast to our data showing an increase in EdU-positive KRas transformed-keratinocytes in the thioredoxin mutant when neutrophils are present (**Figures 5A, B**). Our findings suggest that the increase in transformed cell proliferation in *txn* ^-/-^ larvae requires the presence of neutrophils in the tumor microenvironment.

## Discussion

Here, we report the results of a cell-specific translation profiling screen (TRAP-seq) designed to identify genes differentially expressed in the early tumor microenvironment using the larval zebrafish model. We identified significantly differentially expressed genes on a systemic level in neutrophils and transformed keratinocytes, but not macrophages. One of the most highly upregulated genes in both neutrophils and transformed keratinocytes was the redox regulator and antioxidant thioredoxin. We developed a zebrafish *thioredoxin* mutant line to investigate the role of this antioxidant in the early tumor microenvironment. Our findings support previous studies suggesting that redox regulators are key determinants of inflammation and progression in the tumor microenvironment.

Reactive oxygen species can modulate signaling pathways within the cell (27), as well as act as a chemotactic signal outside of the cell (24). Thioredoxin aids in dampening these ROS levels and thus can influence many signaling pathways in the tumor microenvironment. In our study, with both *in vitro* tissue culture *and in vivo* tissues, depletion of thioredoxin increased ROS signaling. In tumor progression, thioredoxin is typically found to be tumor-promoting (28–30). Our findings support a tumor suppressing role that limits transformed cell growth early in tumorigenesis. Therefore, thioredoxin may have a distinct role during tumor initiation as compared to more developed tumor models used in other studies. Furthermore, our data suggest that the presence of neutrophils in the TME are necessary for thioredoxin to elicit a tumor suppressing effect. A limitation of our study is that we were unable to determine the cell-type specific role of neutrophil thioredoxin on keratinocyte proliferation. Re-expression of cell-specific thioredoxin in *txn* mutant larvae resulted in an inactive protein (data not shown).

Thioredoxin can also alter inflammatory signaling in the tumor microenvironment. We found that expression of the inflammatory cytokine TNFα was increased in transformed cells in the absence of thioredoxin. Our findings are consistent with previous results showing that thioredoxin inhibits NF-κB activation, which drives cytokine production (19). Additionally, exogenous thioredoxin upregulates *tnfα* expression (20). It is possible that the increase in TNFα inhibits neutrophil motility in the TME, as we observed decreased neutrophil speed near transformed keratinocytes. Indeed, previous studies have shown that TNFα induces neutrophil arrest *in vitro* (31). Thioredoxin may also act as a direct neutrophil chemoattractant *in vitro* and *in vivo* (20). However, our own experimental data has shown no evidence of this (data not shown). In fact, there is controversy in the field as thioredoxin has also been shown to protect against neutrophilic inflammation (32). Our data support an inhibitory role for thioredoxin since thioredoxin limited neutrophil recruitment and retention around transformed cells (**Figures 6B, D**). Overall, our data indicate thioredoxin, in the context of early KRas-induced transformation *in vivo*, is inhibitory to inflammatory gene expression and neutrophil motility.

Recent studies have highlighted the importance of understanding neutrophil function in the tumor microenvironment. Many studies have supported a pro-tumor role of neutrophils (4). However how tumor initiation affects neutrophil function remains less clear. Recent work from other cancer models has shown that neutrophils change in the tumor microenvironment and this can be detected by changes in gene expression (33, 34). Our findings suggest that these gene expression changes occur even in the early tumor microenvironment. Thus, oncogenic activation is associated with a systemic shift in neutrophil gene expression, suggesting that neutrophils may provide an early systemic rheostat of tumorigenesis. In the zebrafish model system, we can monitor *in vivo* changes in neutrophil behavior in the tumor microenvironment. Our findings also show that neutrophils are highly dynamic in the early TME, with recruitment very early after oncogenic transformation. Neutrophil migratory behavior changes during the course of tumor development in mouse models, with early stage tumors inducing migration of bone marrow-derived neutrophils and late stage tumors inducing slower, immunosuppressive neutrophil migration (10). Thus, neutrophil motility in the TME can be an indicator of oncogenic activation and tumorigenesis.

In summary, neutrophils in the early tumor microenvironment exhibit changes in their gene expression profile. It remains unclear what specific tumor signals are responsible for inducing these changes in neutrophil gene expression. Our findings suggest that thioredoxin is likely one of many factors that alters neutrophil behavior in the tumor microenvironment. Identifying factors that alter neutrophil behavior will be important for understanding what drives a neutrophil pro-tumor vs anti-tumor phenotype.

## Methods

### Zebrafish Maintenance and *txn* ^-/-^ Line Generation

Zebrafish lines were maintained as previously described (35). To generate *txn* ^-/-^ line, 400pg Cas9 protein (PNA Bio, CP01-50) and 200pg gRNA targeting *txn* exon II were microinjected into one-cell stage NHGRI-1 background. gRNA primers (**Table 1**) and PCR was conducted according to CRISPRscan method (36) and gRNA was synthesized *in vitro* using HiScribe T7 kit (NEB, E2050S) and purified using *mir*Vana miRNA Isolation kit (ThermoFisher, AM1561). CRISPR efficacy in 3dpf F_0_ chimeras was assessed *via* Indel Detection and Amplicon Analysis (IDAA) (40) (**Table 1**). F_0_ chimeras were raised to adulthood and a 1bp insertion was identified *via* IDAA in F_1_ progeny indicating germline mutation of *txn* in the F_0_ generation. The gRNA target region was cloned using the TOPO TA Cloning kit (Life Technologies, 450030) (**Table 1**) and sequenced *via* Sanger sequencing (Functional Biosciences) confirming a 1bp insertion in *txn* exon II. F_1_ *txn* heterozygotes were genotyped *via* PCR (**Table 1**) followed by AlwNI restriction enzyme digestion (NEB, 101229-066). *txn* ^-/-^ and *Tg(LyzC:H2B-mcherry) txn* ^-/-^ lines were generated *via* incrossing and screened using the *txn* genotyping protocol above.

**Table 1:** Primer information.

Species	Primer name	Sequence	Use	Reference
Zebrafish	txn-gRNA1.2-F	taatacgactcactataGGGGGCCCTGCCAGACCATCgttttagagctagaa	CRISPR gRNA generation	–
Zebrafish	gRNA-R constant	AAAAGCACCGACTCGGTGCCACTTTTTCAAGTTGATA ACGGACTAGCCTTATTTTAACTTGCTATTTCTAGCTCTAAAAC	CRISPR gRNA generation	(36)
Zebrafish	M13-txn-F	GTAAAACGACGGCCAGTGGCCTTCGACAACGCCCTAAA	*Txn* genotyping, cloning into TOPO vector, IDAA	–
Zebrafish	txn-R2	AAGCTATTCTCCGCCGGTTG	*Txn* genotyping, cloning into TOPO vector, IDAA	–
Zebrafish	il1b-F	GCCTGTGTGTTTGGGAATCT	RT-qPCR	(6)
Zebrafish	il1b-R	TGATAAACCAACCGGGACAT	RT-qPCR	(6)
Zebrafish	cxcl8-l1-F	GTCGCTGCATTGAAACAGAA	RT-qPCR	(37)
Zebrafish	cxcl8-l1-R	CTTAACCCATGGAGCAGAGG	RT-qPCR	(37)
Zebrafish	cxcl8-l2-F	GCTGGATCACACTGCAGAAA	RT-qPCR	(37)
Zebrafish	cxcl8-l2-R	TGCTGCAAACTTTTCCTTGA	RT-qPCR	(37)
Zebrafish	tnfa-F	GCGCTTTTCTGAATCCTACG	RT-qPCR	(38)
Zebrafish	tnfa-R	TGCCCAGTCTGTCTCCTTCT	RT-qPCR	(38)
Zebrafish	rps11-F	TAAGAAATGCCCCTTCACTG	RT-qPCR	(37)
Zebrafish	rps11-R	GTCTCTTCTCAAAACGGTTG	RT-qPCR	(37)
Human	Luc 1	ACAACTTTACCGACCGCGCC	CRISPR	(39)
	Exon 6		gRNA	
Human	Txn	CCTTTATAAACTGGCACGCC*CGG*	CRISPR	Designed with CRISPOR
	Exon 1		gRNA	
Human	Txn	GAGTCTGACGAGCGGCTGTA*AGG*	CRISPR	Designed with CRISPOR
	Exon 1		gRNA	

To generate an anti-*txn* antibody, full-length zebrafish *txn* was cloned into pTrcHis and expressed in BL21 *E. coli*. His-tagged *txn* was extracted from bacterial lysates using nickel-nitrilotriacetic acid (Ni-NTA) resin (Qiagen, 30410) and subsequently used for anti-sera production in rabbits (Covance). Txn protein depletion was confirmed *via* Western blot using lysates from 30 *txn* ^+/+^ or *txn* ^-/-^ larvae 3dpf. Briefly, larvae were manually deyolked *via* pipette aspiration in Ca^2+^-free Ringer’s solution and lysate generated *via* sonication in lysis buffer (20mM Tris pH 7.6, 0.1% Triton X-100, 0.2mM phenylmethylsulfonyl fluoride (PMSF), 1μg/ml Pepstatin, 2μg/ml Aprotinin, 1μg/ml Leupeptin). Lysates were clarified *via* centrifugation and loaded on a 6-20% SDS-polyacrylamide gel prior to transfer to nitrocellulose and probing with anti-*txn* antisera.

### Plasmid Microinjection

pTol2-krt4-RFP-HRas, pTol2-krt4-RFP-HRas^G12V^, and pTol2-krt4-L10a-EGFP constructs used in TRAP RNA-sequencing experiment were generated as previously described (7). pTol2-krtt1c19e-KRas^G12V^-mcherry, pTol2-krtt1c19e-KRas^G12V^-GFP, and pTol2-krtt1c19e-L10a-GFP were cloned *via* PCR of inserts containing the gene of interest and homologous recombination into pTol2 vectors using the In-Fusion HD cloning kit (Clontech, 638911). All constructs microinjected contained Tol2 transposable elements thus allowing incorporation of the relevant promoter and gene-of-interest into the zebrafish genome (41).

Embryos at 1-cell stage were microinjected as previously described (7) with 3nl injection mix containing 37.5ng relevant plasmid, indicated in figure legend, and 52.5ng *tol2* mRNA. Injected embryos were incubated at 28.5°C in E3 media (5mM NaCl, 0.17mM KCl, 0.33mM CaCl_2_, 0.33mM MgSO_4_) containing 0.2mM N-phenylthiourea (PTU) to inhibit pigment formation (Sigma, P7629). Larvae were anesthetized with 0.2mg/ml Tricaine (Pentair, trs1) in E3 3dpf and screened for transgene expression using Zeiss Stereo Zoom Microscope (EMS3/SyCoP3; Plan-NeoFluar Z objective).

### Translating Ribosome Affinity Purification

Larvae were microinjected and screened 3dpf for transgene expression as described above. Microinjections for TRAP-RNA-sequencing included either control pTol2-krt4-RFP-HRas or oncogenic pTol2-krt4-RFP-HRas^G12V^ constructs injected with pTol2-krt4-GFP-L10a in wildtype larvae, or into *Tg(LyzC : EGFP-L10a)* or *Tg(mpeg1:EGFP-L10a)* larvae (13) for keratinocyte, neutrophil, or macrophage-specific expression profiling, respectively. For TRAP-qPCR experiments, control pTol2-krtt1c19e-mcherry-KRas or oncogenic pTol2-krtt1c19e-mcherry-KRas^G12V^ was injected into *txn* ^+/+^ or *txn* ^-/-^ larvae in conjunction with the TRAP construct pTol2-krtt1c19e-EGFP-L10a. Following microinjection, larvae were screened for transgene expression 3dpf and subsequently stored at -80°C in minimal residual media. The TRAP lysis and ribosome immunoprecipitation procedure was conducted as previously reported (13) using 50 larvae per condition per replicate. Immunoprecipitated mRNA was extracted from polysomes following Trizol (ThermoFisher, 15596026) manufacturer recommendations. 70% ethanol was added to the aqueous layer at 1:1 ratio and mRNA was purified using the RNAqueous Micro kit (ThermoFisher, AM1931).

### RNA Sequencing and Analysis

cDNA libraries were generated from aforementioned TRAP samples as previously described (16) and sequenced on an Illumina HiSeq system. An average of 23 million single-end reads were generated and aligned to zebrafish reference genome GRCz10 using Bowtie v1.1.1 (42). Transcript abundance was quantified using RSEM v1.2.20 (43) and differential expression between conditions was assessed using DESeq2 (44). To control for possible batch effects, the design formula “ ~ replicate + condition” was used for DESeq2’s generalized linear model, where “condition” was the combination of cell type and treatment (control or oncogenic construct) for each sample. Statistical testing for differential expression within each cell type was performed using the Wald test implemented in the DESeq2 package and genes with a Benjamini–Hochberg corrected P value (FDR) ≤ 0.05 were considered statistically significant. Zebrafish genes were matched to human orthologs using BioMart and Hallmark gene set differences assessed *via* Gene Set Enrichment Analysis (45). *Txn* transcript levels in human tumors samples from The Cancer Genome Atlas (TCGA) were assessed using Firebrowse (http://firebrowse.org/).

### qPCR

mRNA isolated *via* TRAP was DNAse treated (Promega, M6101) for 30 min at 37°C. cDNA was synthesized using SuperScript III First-Strand Synthesis kit (ThermoFisher, 18080051) according to manufacturer specifications. Sybr green (Roche) qPCR master mix was used to quantify *il1b*, *cxcl8a*, *cxcl8b.1*, and *tnfa* gene expression in addition to *rps11* expression as a normalization control (**Table 1**). qPCR reactions were run on a Lightcycler (Roche) and Cq values were quantified from Lightcycler software and normalized to housekeeping gene *rps11*.

### Neutrophil Migration Movies

Neutrophil behavior in response to basal transformation was carried out in LyzC-H2B-mcherry *txn* mutant lines. These lines were injected with pTol2-krtt1c19e-KRasG12V-GFP as described. Locations with 30-40 transformed cells within the FOV of 40x objective were selected. Timelapse images were taken at 90 second intervals for 2.5 hours on a Zeiss spinning disk confocal (Yokogawa, CSU-X) microscope with Photometrics Evolve EMCCD camera. Neutrophils were tracked with Imaris analysis software and mean track speed was calculated.

Neutrophil random migration was assessed in unstimulated LyzC-H2B-mcherry *txn* wildtype and mutant lines. Timelapse images were taken at 90 second intervals for 2.5 hours using a 10x objective on a Zeiss spinning disk confocal (Yokogawa, CSU-X) microscope with Photometrics Evolve EMCCD camera. Neutrophils were tracked with Imaris software and mean track speed was calculated.

### Tail Transection Assays – pfbs-f ROS Probe and Neutrophil Recruitment

Hydrogen peroxide abundance was assessed with the probe pentafluorobenzenesulfonyl fluorescein (pfbs-f) (Santa Cruz, sc-205429). Tail transection was conducted 3dpf (**Figure 2C**) with a sterile scalpel blade in a solution of 1uM pfbs-f and 0.2mg/ml Tricaine in E3. 30 minutes post-wound (mpw) and 4 hours post-wound (hpw), transected tails were imaged at 20x magnification *via* spinning disk confocal microscopy (Yokogawa, CSU-X). Pfbs-f mean gray value was quantified from maximum intensity projections in an outlined region-of-interest posterior to the notochord extending to and encompassing the wound margin.

To assess neutrophil abundance, *Tg(LyzC:H2B-mcherry) txn* ^+/+^ and *Tg(LyzC:H2B-mcherry) txn* ^-/-^ larvae 3dpf were wounded as above in 0.2mg/ml Tricaine in E3. Neutrophil abundance posterior to the notochord was assessed 2hpw and 6hpw *via* spinning disk confocal microscopy (Yokogawa, CSU-X) at 20x magnification.

### HL60 Cell Culture

HL-60 cells were maintained in RPMI 1640 1X with l-glutamine and 25 mM HEPES (Corning 10-041-CV) supplemented with 10% heat-inactivated fetal bovine serum (FBS; HyClone, SH30071.03) and 1X penicillin–streptomycin (Corning; 30-002-CI) at 37°C, 5% CO_2_.

All HL60 cells were differentiated in RPMI 1640 complete media supplemented with 1.3% DMSO (Sigma-Aldrich; D2650) at a density of (3–4) × 10^5^ cells/ml in 10 ml for 6 d at 37°C, 5% CO_2._

### Thioredoxin Knockdown in HL60 Neutrophil-Like Cells

Guide RNAs (gRNAs) were designed against early exons of target genes using the Crispor tool (46) (**Table 1**). The gRNAs with highest specificity and lowest off-target likelihood were chosen and synthesized by IDT as Alt-R synthetic single guide RNA (sgRNA). For generation of thioredoxin knockout cell lines, two individual gRNAs targeting the same exon about 100 base pairs apart were simultaneously transfected. gRNAs targeting the Luciferase gene were utilized as a control. HL60 cells were transfected with ribonucleoprotein (RNP) complexes, FACS sorted, and expanded as previously described (47).

Western blotting was conducted to confirm protein knockdown in the generated HL60 cell lines. Cell pellets were collected and lysed in 1X RIPA buffer (50 mM Tris-HCl, pH 8.0, 150 mM NaCl, 1% NP-40, 0.5% sodium deoxycholate, 0.1% SDS) with 1X Halt Protease and Phosphatase Inhibitor Cocktail (Thermo Scientific; 78840) for 30 min on ice. Lysates were sonicated for three cycles at 20% amplitude (5 s on, 10 s off) and cleared by centrifugation at 15,000 × *g*, 4°C for 15 min. Protein concentrations were determined using the Pierce BCA Protein Assay (Thermo Scientific; 23225) and samples stored at −80°C. Protein (20–30 µg) was heated in 4X Bolt LDS Sample Buffer (Invitrogen; B0007) with 10X Bolt Sample Reducing Agent (Invitrogen; B0004) for 10 min at 70°C. Samples were loaded into precast Bolt 4–12% Bis-Tris Plus Gels (Invitrogen) and run for 35 min at 200 V in 1X Bolt MES SDS Running Buffer (Invitrogen; B0002). Proteins were transferred onto nitrocellulose membranes for 60 min at 90 V. Membranes blocked with 5% milk in TBS-0.1% Tween 20 (TBS-T) for 1 h at RT. Membranes were washed and inverted onto primary Txn antibody (Sigma, SAB1409783) in 5% milk TBS-T overnight at 4°C. After washing, the membranes were incubated in anti-mouse secondary antibody (Invitrogen, A-21131) for 60 min at RT. Membranes were washed and scanned on an Odyssey scanner (LICOR) at 700 and 800 nm wavelengths. Images were analyzed in Image Studio software (LICOR).

### *In Vitro* Neutrophil Assays- Reactive Oxygen Species Production and Chemotaxis

Intracellular ROS production was quantified using the ROS indicator Carboxy-H2DCFDA (Invitrogen, C400). HL60 cells were differentiated as described above, then re-suspended in 0.5%HSA-PBS for 2.5 hours to serum starve. Cells were incubated for 30 minutes at 37°C in PBS containing 20uM Carboxy-H2DCFDA, spun down, and re-suspended in 0.5%-HSA-HBSS. Cells were plated into a fibronectin coated (10ug/mL) black 96-well plate at 100k cells per well. A 2X PMA solution was added to each well to achieve a final concentration of 1ng/mL PMA. Fluorescence was read every 30 minutes for two hours using the Victor3V microplate reader using the 485nm/535nm filter (PerkinElmer).

Extracellular H_2_O_2_ production was assessed using the Amplex Red/HRP probe (Invitrogen, A22188) as previously described (48). Briefly, 100uL of the reaction mixture containing 50uM Amplex Red and 0.1U/mL HRP was added to each well of a white 96-well plate coated with fibronectin (10ug/mL). The plate was incubated for at least 10 minutes at 37°C. Differentiated HL60 cells were re-suspended in Krebs-Ringer buffer at 750k/mL and plated at 20uL/well. Absorbance was read every hour for 3 hours at 570nm using the Victor3V microplate reader (PerkinElmer).

Chemotaxis was assessed using a microfluidic device as described previously (49). In brief, polydimethylsiloxane devices were plasma treated and adhered to glass coverslips. Devices were coated with 10 μg/mL fibrinogen (Sigma) in PBS for 30 min at 37°C, 5% CO_2_. The devices were blocked with 2% BSA-PBS for 30 min at 37°C, 5% CO_2_, to block non-specific binding, and then washed twice with mHBSS. Cells were stained with calcein AM (Molecular Probes) in PBS for 10 min at room temperature followed by resuspension in modified Hank’s balanced salt solution (mHBSS). Cells were seeded at 5 × 10^6^/mL to allow adherence for 30 min before addition of chemoattractant. Then, 1 μM fMLP (Sigma) was loaded onto the devices. Cells were imaged for 45–90 min every 30 s on a Nikon Eclipse TE300 inverted fluorescent microscope with a 10× objective and an automated stage using MetaMorph software (Molecular Devices). Automated cell tracking analysis was done using JEX software (50) to calculate chemotactic index and velocity.

### EdU Proliferation Assay and Immunofluorescence

pTol2-krtt1c19e-KRas^G12V^ injected *txn* ^+/+^ or *txn* ^-/-^ larvae were treated with 250uM EdU (ThermoFisher, C10338) for 6.5hrs at 28°C in E3 + PTU 3dpf. Larvae were fixed in formaldehyde buffer (100mM PIPES, 1mM MgSO_4_, 2mM EGTA, 1.5% formaldehyde, 0.2mg/ml Tricaine) in E3 and stored at 4°C. Fixation buffer was exchanged for methanol and larvae were placed at -20°C overnight. Samples were rehydrated in a sequence of methanol dilutions (75% methanol:25% PBSTx (0.2% Triton X-100), 50% methanol:50% PBSTx, 25% methanol:75% PBSTx), washed in PBSTx, and blocked for 3 hours in blocking buffer (1.5% v/v sheep serum, 1% w/v BSA, 0.2% Triton X-100 in PBS) mixing *via* nutator. Alexa Fluor 555 azide conjugation was conducted according to Click-it manufacturer specifications (ThermoFisher, C10338) in 350μl at 4°C overnight on rocker. Antibody staining was utilized to allow for visualization of GFP-Kras-expressing cells. Samples were washed twice in PBSTx and blocked once in blocking buffer prior to primary rabbit α-GFP Ab treatment (ThermoFisher, A11122). Primary antibody treatment was incubated in 350μl at 1:200 dilution in blocking buffer for 4 hours at room temperature. Samples were washed twice in PBSTx, twice in blocking buffer and treated with secondary donkey α-rabbit AF488 (Jackson ImmunoResearch, 711-545-152) in 350ul at 1:250 dilution in blocking buffer overnight at 4°C. Samples were washed 6 times in PBSTx prior to imaging *via* confocal microscopy. EdU-positive cells were manually quantified in FIJI or Imaris.

Active caspase-3 staining was conducted as above, omitting EdU and Alexa Fluor 555 azide treatment. Primary antibody staining utilized rabbit α-active caspase-3 (BD Pharmingen, 559565) at 1:300 dilution and secondary staining used donkey α-rabbit Dylight 405. Images were obtained *via* confocal microscopy and intracellular active caspase-3 mean gray value was calculated in FIJI.

### Statistical Analysis

Statistical analyses were performed on at least three independent experimental replicates, unless otherwise indicated in the figure legend. For all experiments except neutrophil speed assays, data points represent measurements from individual larvae. Replicate number is distinguished by differential data point color and shape, with the mean replicate value presented in bold. For neutrophil speed experiments, data points represent values from a given neutrophil, whereas bolded data points display the average neutrophil speed for a given larvae. Data were tested for normality with Shapiro-Wilk test (p<0.05). Statistical significance (p<0.05) was calculated by t-test or Mann-Whitney U test for normally or non-normally distributed data, respectively, in R version 3.6 and graphed in GraphPad Prism version 9 using measurements from individual larvae as the sampling unit.

For *in vitro* quantification of ROS production, statistical analyses were performed using a generalized estimating equation (GEE) fit to the log background corrected fold change to estimate the effect associated with each Txn-knockdown cell line relative to the control. Model assumed Gaussian distribution and were analyzed using R v3.6.2.3 and the associated *geepack* package. Statistical significance was pre-defined as p< 0.05 with confidence intervals set to 95% coverage. For *in vitro* cell tracking experiments, statistical significance (p<0.5) was determined by one-way ANOVA in GraphPad Prism version 9.

## Data Availability Statement

The data presented in the study are deposited in the GEO repository, accession number GSE193591.

## Ethics Statement

The animal study was reviewed and approved by Institutional Animal Care and Use Committee of University of Wisconsin.

## Author Contributions

BK and AH conceived and designed experiments. BK, MG, GR, CD, DB, JR, SM, and AH conducted the experiments and analysis. AH, MG, and BK prepared the figures and wrote the manuscript. All authors contributed to the article and approved the submitted version.

## Funding

The authors would like to acknowledge NIH/NCI R01 CA085862 to AH and T32AI055397 to MG.

## Conflict of Interest

The authors declare that the research was conducted in the absence of any commercial or financial relationships that could be construed as a potential conflict of interest.

## Publisher’s Note

All claims expressed in this article are solely those of the authors and do not necessarily represent those of their affiliated organizations, or those of the publisher, the editors and the reviewers. Any product that may be evaluated in this article, or claim that may be made by its manufacturer, is not guaranteed or endorsed by the publisher.

## Supplementary Material

Supplementary Figure 1 Expression of *txn* in human tumors compared to healthy tissue. **(A)** Hierarchical clustering of Gene Set Enrichment Analysis (GSEA) of enriched Hallmark pathways in neutrophils, keratinocytes, and macrophages for HRas^G12V^-keratinocyte expressing zebrafish. **(B)** mRNA expression profile of *txn* in human cancer and normal tissue. Firebrowse was used to extract expression profiles of human tumors from The Cancer Genome Atlas (TCGA).

Supplementary Figure 2
*Txn* mutants display no basal increase in neutrophil number or inflammatory gene expression. **(A)** Neutrophil distribution and abundance in unstimulated *txn* ^+/+^ Tg(*LyzC*:*H2B-mcherry*) or *txn* ^-/-^Tg(*LyzC*:*H2B-mcherry*) larvae 3dpf. **(B)** Quantification of total neutrophil number in unstimulated larvae, wild-type (n=41) and mutant (n=40). **(C)** qPCR of inflammatory cytokines *il1b*, *cxcl8a*, *cxcl8b.1*, *tnfa* in whole larval lysates (pooled, 30 larvae) from *txn* wild-type (n=2) or mutant (n=2) larvae 3dpf. Large, bolded shapes indicate average value per replicate (n), with small shapes representing data points from independent larvae. Samples were analyzed for statistical significance *via* t-test (p<0.05*, p<0.01**, p<0.001***, p<0.0001****).

Supplementary Figure 3 Neutrophil random migration is unaffected in the *txn* mutant. **(A)** Neutrophil random migration speed in the head region of unstimulated *txn* ^+/+^ Tg(*LyzC*:*H2B-mcherry*) or *txn* ^-/-^Tg(*LyzC*:*H2B-mcherry*) larvae 3dpf. Track color indicates average velocity per neutrophil tracked. **(B)** Quantification of average neutrophil speed in *txn* wt (n=4) and *txn* mut (n=4) larvae. Scale bar, 10um. Large, bolded shapes indicate average value per replicate (n), with small shapes representing data points from independent larvae. Samples were analyzed for statistical significance *via* t-test (p<0.05 *, p<0.01**, p<0.001***, p<0.0001****).

Supplementary Figure 4 Schematic of basal keratinocyte KRas^G12V^ zebrafish model. **(A)** Schematic of microinjection procedure. One cell stage *txn* ^+/+^ Tg(*LyzC*:*H2B-mcherry*) or *txn* ^-/-^Tg(*LyzC*:*H2B-mcherry*) embryos are injected with basal keratinocyte transformation constructs (pTol2-krtt1c19e-KRas^G12V^-mcherry).
