# Supplementary figures and images for: Cell Type-Specific Transcriptome Profiling Reveals a Role for Thioredoxin During Tumor Initiation
Source: Front Immunol. 2022 Feb 17;13:818893. doi: 10.3389/fimmu.2022.818893 (PMC8891495; doi:10.3389/fimmu.2022.818893)

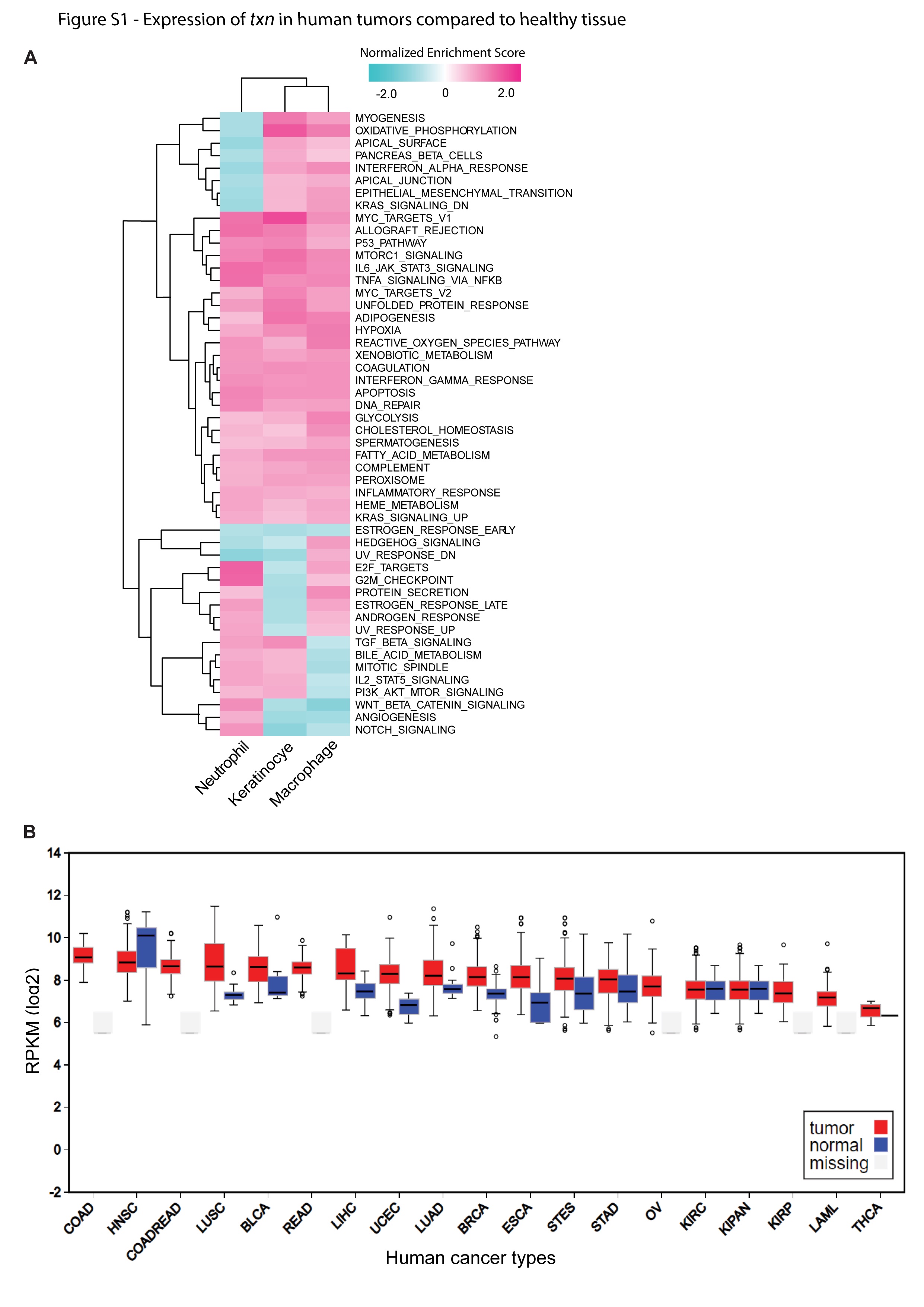

Supplement: Supplementary Figure 1 — Expression of txn in human tumors compared to healthy tissue. (A) Hierarchical clustering of Gene Set Enrichment Analysis (GSEA) of enriched Hallmark pathways in neutrophils, keratinocytes, and macrophages for HRasG12V-keratinocyte expressing zebrafish. (B) mRNA expression profile of txn in human cancer and normal tissue. Firebrowse was used to extract expression profiles of human tumors from The Cancer Genome Atlas (TCGA). [file Image_1.jpg]

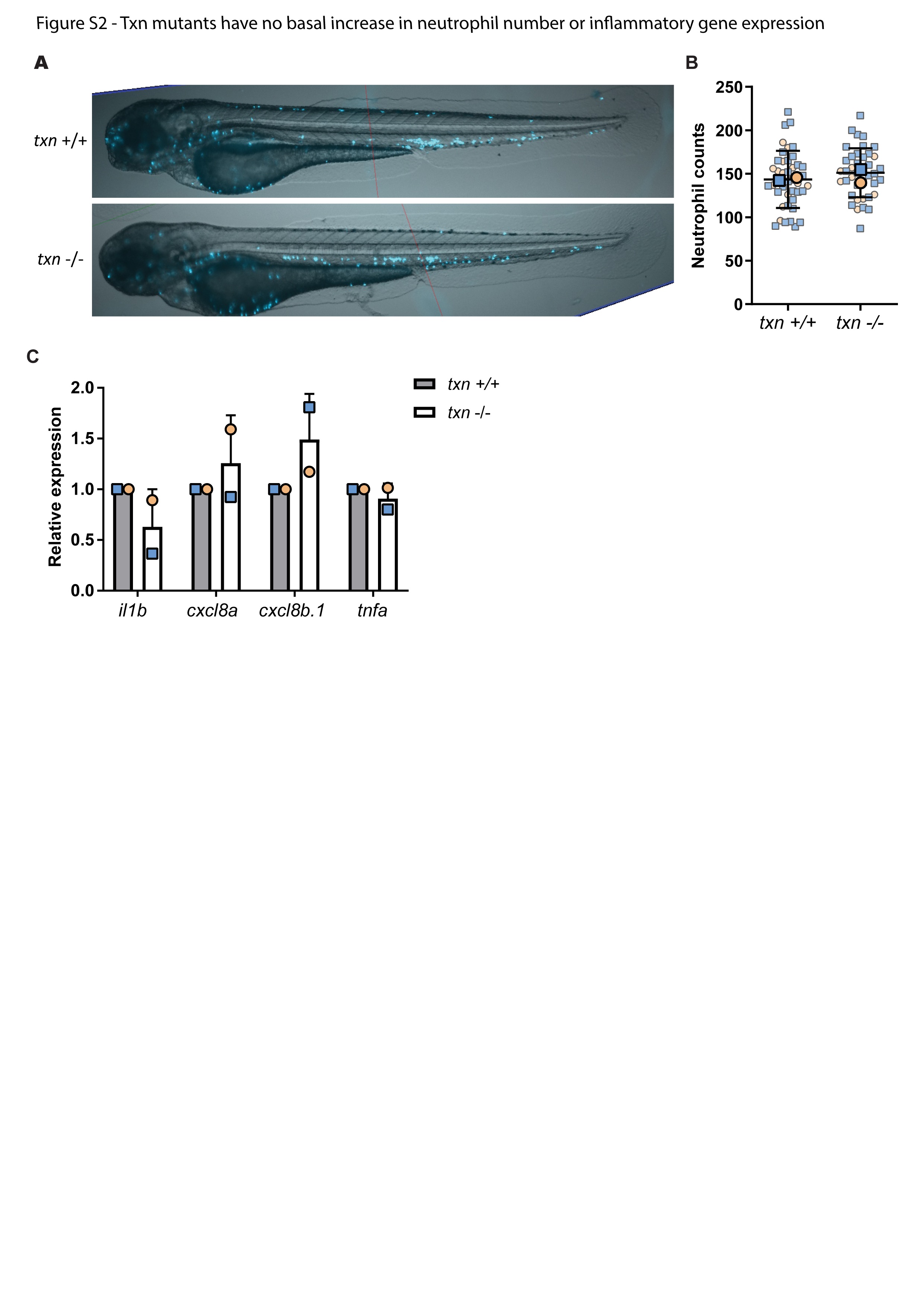

Supplement: Supplementary Figure 2 — Txn mutants display no basal increase in neutrophil number or inflammatory gene expression. (A) Neutrophil distribution and abundance in unstimulated txn +/+ Tg(LyzC:H2B-mcherry) or txn -/-Tg(LyzC:H2B-mcherry) larvae 3dpf. (B) Quantification of total neutrophil number in unstimulated larvae, wild-type (n=41) and mutant (n=40). (C) qPCR of inflammatory cytokines il1b, cxcl8a, cxcl8b.1, tnfa in whole larval lysates (pooled, 30 larvae) from txn wild-type (n=2) or mutant (n=2) larvae 3dpf. Large, bolded shapes indicate average value per replicate (n), with small shapes representing data points from independent larvae. Samples were analyzed for statistical significance via t-test (p<0.05*, p<0.01**, p<0.001***, p<0.0001****). [file Image_2.jpg]

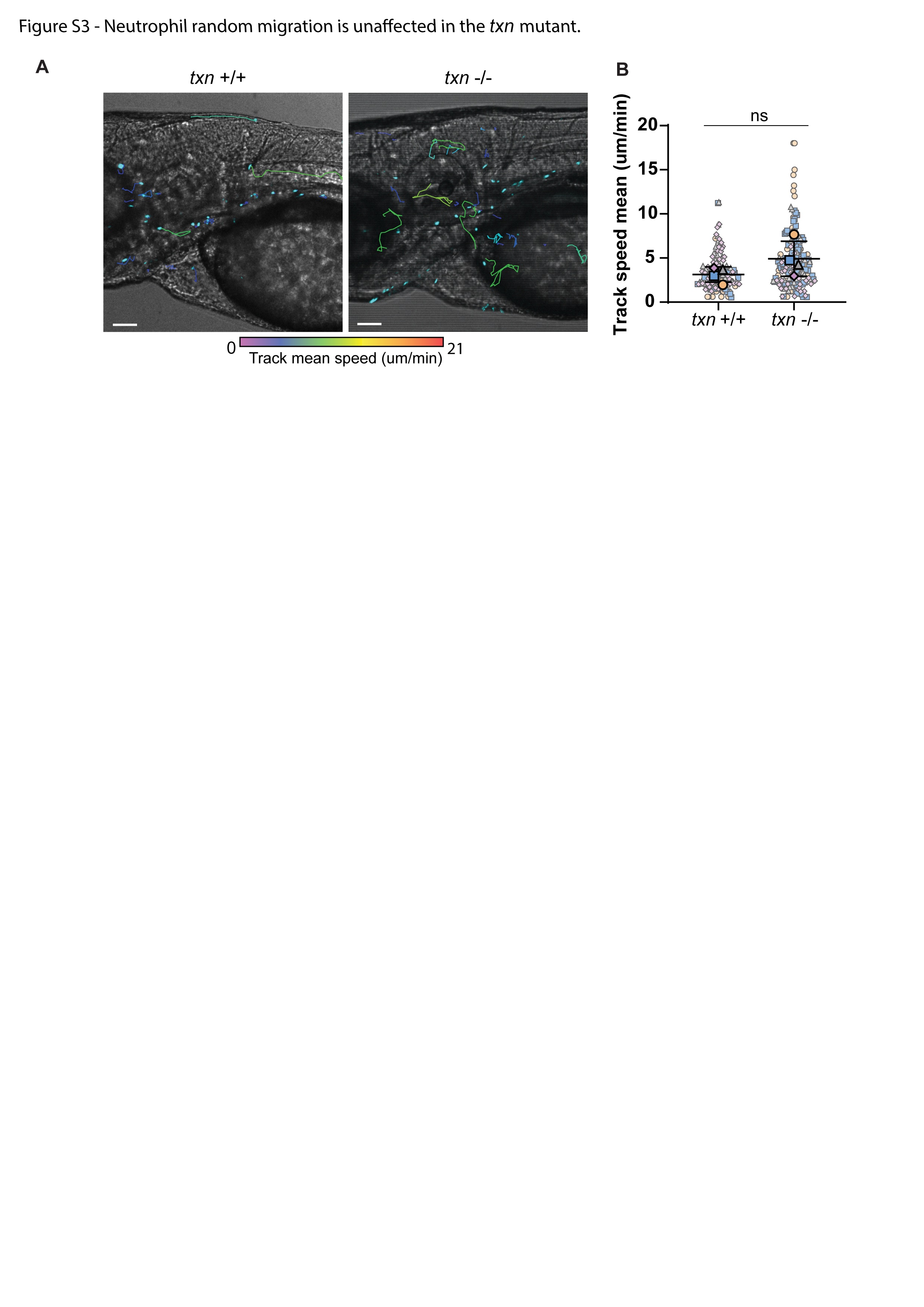

Supplement: Supplementary Figure 3 — Neutrophil random migration is unaffected in the txn mutant. (A) Neutrophil random migration speed in the head region of unstimulated txn +/+ Tg(LyzC:H2B-mcherry) or txn -/-Tg(LyzC:H2B-mcherry) larvae 3dpf. Track color indicates average velocity per neutrophil tracked. (B) Quantification of average neutrophil speed in txn wt (n=4) and txn mut (n=4) larvae. Scale bar, 10um. Large, bolded shapes indicate average value per replicate (n), with small shapes representing data points from independent larvae. Samples were analyzed for statistical significance via t-test (p<0.05 *, p<0.01**, p<0.001***, p<0.0001****). [file Image_3.jpg]

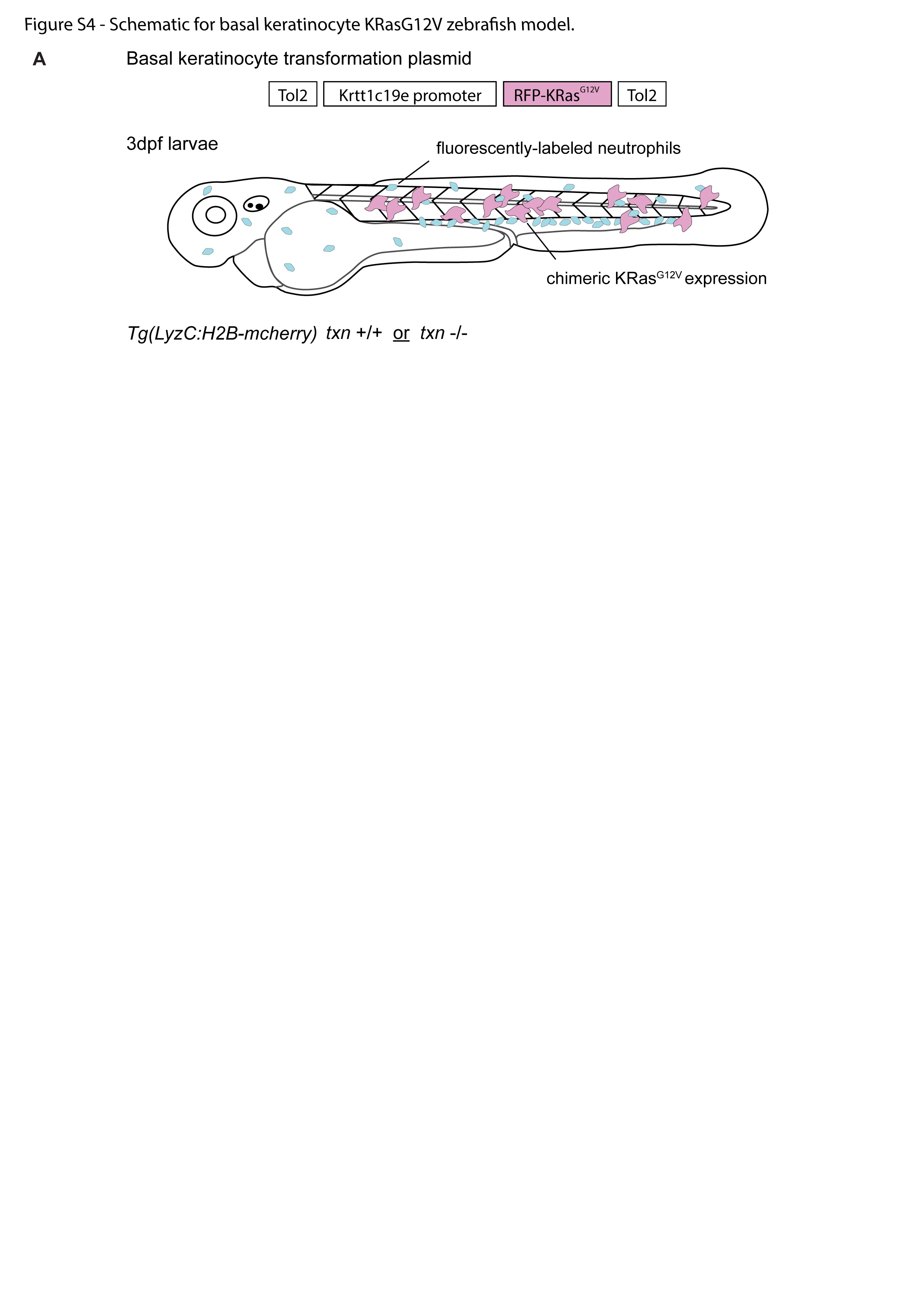

Supplement: Supplementary Figure 4 — Schematic of basal keratinocyte KRasG12V zebrafish model. (A) Schematic of microinjection procedure. One cell stage txn +/+ Tg(LyzC:H2B-mcherry) or txn -/-Tg(LyzC:H2B-mcherry) embryos are injected with basal keratinocyte transformation constructs (pTol2-krtt1c19e-KRasG12V-mcherry). [file Image_4.jpg]
